# The Influences of Chloride of Sodium on Absorption and Exhalation

**Published:** 1854-02

**Authors:** Henry Parker


*The Influences of Chloride of Sodium on Absorption and Exhalation; a paper read before the Cook County Medical Society*, *by* Henry Parker, *M.D.*

Among the most important remedial agents having a special influence upon the processes of absorption and exhalation, and the due interchange of these elements, I may mention Chloride of Sodium.

This substance, as the Society is well aware, is attracting the attention of medical men, as a remedy possessing peculiar and important powers in the treatment of certain forms and conditions of diseased action.

That a substance of such free and common use should possess the influences ascribed to it, may naturally excite the surprise and distrust of the ignorant and the charlatan; but the man of science, who sees in disease the absence of some element material and essential to the healthy body, or the presence of one unnatural and pernicious, will, when acquainted with its *modus operandi*, readily understand how it accomplishes certain good.

From some recent experiments, made by M. Bernard, of Paris, upon the effect of this salt upon the blood, he ascertained that it greatly increased the capacity of this fluid for the absorption of oxygen.

*Experiment.*—Into two graduated glass tubes, containing each equal quantities of pure oxygen gas, he introduced equal quantities of blood freshly drawn from the jugular vein of a dog, without being exposed to external air. One of these tubes contained a small quantity of chloride of sodium. The blood in this tube absorbed immediately thirty-two parts of oxygen, while the blood contained in the tube, with only pure oxygen, absorbed only twenty parts.

Observe what a difference in the relative amounts of oxygen absorbed.

As this experiment of M. Bernard was made upon blood out of the body, and without any special reference to the proportionate amount of carbonic acid expired, I was desirous of ascertaining how far the internal administration of the salt, would, in its influences upon absorption, affect the exhalation of this excretion, carbonic acid.

With this view, I confined myself for a few days to a diet, plain, simple, and as free from *salt* as could well be obtained. During this interval, a given quantity of expired air was obtained from time to time, in a graduated glass tube of known capacity, over mercury, and transferred to a solution of caustic potassa.

The results of these repeated experiments may be expressed thus: Whole amount of carbonic acid contained in each given quantity of expired air as indicated by the rise in the tube, 1.25 of a cubic inch.

The experiments were continued sufficiently long to furnish a reliable data, with which to compare future results.

Immediately upon concluding the above experiments, I took liberally of common salt, and especially upon going to bed at night.

The amount of carbonic acid contained in the same given quantity of expired air, obtained the following morning, and at the same hour as in some of the preceding experiments, viz. immediately upon rising from bed, was

Carbonic acid, 3.32 cubic inch.
Sp. G. of Urine, 1.035.

This experiment was repeated several times, observing the same diet, and, as in the former experiments, with this difference only, that I partook freely of salt, and with the same results as expressed above.

Observe what a material difference in the relative amounts of carbonic acid exhaled in each given quantity of expired air, as obtained from these two series of experiments—an increase in the latter over the former of one-hundred-and-thirty per cent.

It will be observed that these inquiries were made with special and only reference to the amount of carbonic acid exhaled, and not with any reference to the quantity of oxygen absorbed.

It is very probable, however, that in the process of absorption and exhalation, these two gases sustain certain definite relations with each other. What the proportion which exists between them may be, is not definitely determined. But that the increased absorption of one is attended by a corresponding increase in the exhalation of the other, I think is true. Blood may contain various gases; but it is evident that it never disengages them spontaneously. "They must always be extracted by displacement, in submitting to it some other gas, for which it may have a stronger affinity. This general rule is applicable to liquids,—these can only lose one of their component elements, by receiving a different one. Exosmose and endosmose, must occur." Bernard's experiment tends to establish the correctness of this, when applied to the animal economy. Beneath the skin of a rabbit he injected a quantity of air. In due time this was analyzed, and found not to have diminished materially in quantity, but to have undergone a change. The oxygen only had been absorbed, and carbonic acid occupied its place. From these facts it is very evident that the entrance of one gas into the system is always attended by the corresponding exit of another. Hence the increased exhalation of carbonic acid observed in some of the above experiments—the result of an increased absorption of oxygen.

From the preceding inquiries into the influence of chloride of sodium upon the blood, we observed three important facts:

1st. An increase in the capacity of this fluid for the absorption of oxygen.

2d. An increase in the amount of carbonic acid exhaled. And,

3d. An augmented excretion of urea.

The effects observed upon these several processes are so marked that every intelligent and scientific physician must see the importance of their due consideration, in the treatment of certain forms of diseased action. With these facts before us, we infer that the use of this substance is indicated in those diseases, dependent upon, or accompanied by, a too small supply of the former, and an undue accumulation and retention in the system of the latter.

Does experience confirm this view, and the results of its use in such cases establish the correctness of our deductions? If we call to mind the pathology of those diseases, in which its use has been attended with the most marked benefit, we observe the existence of one or all of these three conditions, viz:

A diminished supply of oxygen, with an abnormal accumulation and retention in the system of carbonic acid and urea.

Congestive pneumonia affords us a good illustration of this,—a want of efficient oxygenation of the blood, and the due elimination of carbonic acid and those morbid substances designed for excretion. This want, oftentimes, if not generally, constitutes the only real source of danger to the welfare of the patient. That other means and other remedies may be demanded to subdue the local trouble, to remove the cause, of which this is an effect, I admit; but the period of time required to effect this is often protracted,—the patient sinking from a want of efficient oxygenation of the blood. This fact indicates the use of such remedy, combined with those adapted to the removal of the cause, as will most effectually promote the due interchange of these elements, viz. oxygen and carbonic acid.

Reasoning then from the known effect of chloride of sodium upon the blood, in promoting the absorption of oxygen and alimination of carbonic acid, we infer that, in this substance, do we find such a remedy.

And the fact, also, that the greatest apparent good results from its use, when such pathological conditions exist, it seems to me, furnishes strong evidence that its *modus operandi*, or rather, *modus curandi*, is through its influences upon absorption and exhalation.

Does the experience of the Society in the use of this substance confirm this view?

In this connection, also, I might mention, as illustrating farther the *modus curandi* of the remedy under consideration, that class of diseases having a malarious origin, as exhibited in intermittent and remittent fevers. It is not my purpose now to attempt any explanation, or advance any theory relative to the exciting cause of these affections, further than to call to mind what has been advocated by the President of this Society and concurred in by others, viz:

That an important element, concerned in the production and development of miasmatic diseases, is "an atmosphere saturated with moisture, and constituted in part of carbonic acid, sulphuretted and carbonetted hydrogen, and other gases, such as deteriorate the air, both by their poisonous qualities and by their affinity for oxygen;" in a word, there is "defective oxygenation and an excess of carbonaceous material in the blood and tissues."

Now, if this be true, we can readily understand something of the *modus curandi* of chloride of sodium, when used in this class of affections. That it will arrest the paroxysms of an intermittent or remittent fever quite as effectually and promptly as quinine, all, I think, who have had experience with it as a remedy in such cases, will admit. Whence, then, this power? What the *rationale* of its operations? Evidently, is it not through its influence principally upon the blood, in promoting the ready absorption of oxygen, and the free elimination of those substances having a pernicious effect upon the economy? That it may exert *other* influences, and induce other and important changes in the system, all tending to bring about the same beneficial results, is unquestionably true, but as I purposed only to present a few facts relative to its importance as a therapeutic agent in promoting absorption and exhalation, I omit any further consideration of its *modus operandi* not immediately influencing this function.

Much additional evidence might be adduced in further support of the views presented, but as briefness in *society papers* oftentimes constitutes one of their *greatest* merits, if not their only redeeming quality, I submit the subject without further comment, asking for the facts presented and the deductions made, only that candid consideration from the Society which their value or importance may merit.—*N. W. Med. and Surg. Jour.*

